# An instantaneous ECV with no blood sampling: using native blood T1 for hematocrit is as good as standard ECV

**DOI:** 10.1186/1532-429X-17-S1-Q129

**Published:** 2015-02-03

**Authors:** Thomas A Treibel, Arthur Nasis, Marianna Fontana, Viviana Maestrini, Silvia Castelletti, Anish N Bhuva, Stefania Rosmini, Amna Abdel-Gadir, Heerajnarain Bulluck, Peter Kellman, Stefan K Piechnik, Matthew D Robson, James Moon

**Affiliations:** 1 The Heart Hospital Imaging Centre, University College London, London, UK; 2 Oxford Centre for Clinical Magnetic Resonance Research, Oxford, UK; 3 National Heart, Lung and Blood Institute, Bethesda, MD, USA; 4 Department of Cardiovascular, Respiratory, Nephrology and Geriatrics Sciences, La Sapienza, University of Rome, Rome, Italy; 5 Monash Cardiovascular Research Centre, Sydney, NSW, Australia

## Background

The extracellular volume fraction (ECV) by T1 mapping measures the size of the myocardial interstitium. T1 changes in blood and myocardium are used to measure the contrast partition coefficient (λ), and substituting in the blood volume of distribution (directly measured on a peripheral blood sample as one minus the hematocrit [Hct]) provides the ECV. This methodology is however cumbersome, has significant variability, introduces a delay and is a barrier to wider use of ECV quantification in clinical practice. We have previously observed a strong relationship between ShMOLLI T1_blood_ and Hct [Piechnik, JCMR 2013, 15:13] and hypothesise that this could be used to infer the Hct at the time of scan and permit immediate ECV calculation without blood sampling (ECV_No Hct_).

## Methods

350 subjects (age 61±15 years; 47% male; 36 healthy volunteers, 95 severe aortic stenosis, 95 with a history of anthracycline chemotherapy, 46 hypertrophic cardiomyopathy, and 78 cardiac amyloidosis) underwent T1 mapping with ShMOLLI at 1.5T (Siemens Avanto) prior to and at 15 minutes after administration of 0.1mmol/kg of Dotarem. Venous blood for Hct was obtained prior to scanning. The partition coefficient λ = (Δ[1/T1_myo_] / Δ[1/T1_blood_]) and ECV_Hct_ = λ * [1-haematocrit]) were calculated. Hct was approximated from the linear relationship with native T1_blood_ and used to calculate ECV_No Hct_. This was then compared to the conventional ECV_Hct_, partition coefficient and post-contrast T1_myocardium_.

## Results

**Figure 1:**
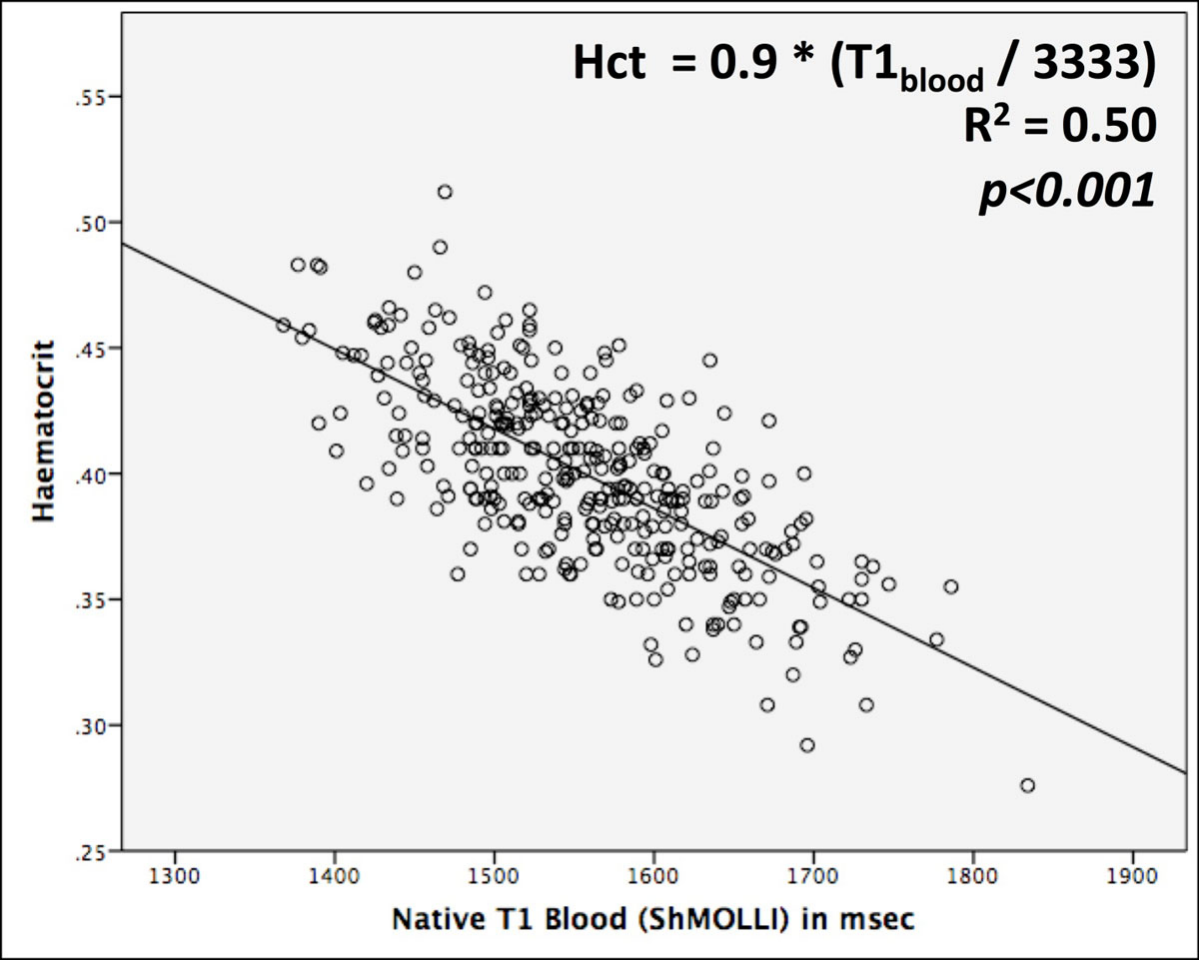
Correlation between hematocrit and native T1_blood_.

There was strong correlation between ShMOLLI T1_blood_ and Hct across health and disease with a coefficient of explained variation R^2^=0.50 (*p*<*0.001;* Figure 1), i.e. 50% variability of native T1_blood_ apportioned to the Hct. The broad array of cardiac pathologies provided a wide range of Hct (40.0±3.6%; range 28-51%) and native T1_blood_ (1557±81ms; range 1368-1834ms), with similar correlations of Hct versus T1_blood_ in each group. The regression equation was: Hct = 0.9 - (T1_blood_ / 3333).

Derived ECV_No Hct_ exhibited excellent correlation with conventional ECV_Hct_ (R^2^=0.99; *p*<*0.001*) with small ~2% bias and ~3% SD of differences on Bland-Altman analysis (95% confidence interval -0.7 to +3.9% excluding Amyloid, and -2.6 to +8.0% for Amyloid) close to previously reported 1.4% [Schelbert EB JCMR 2011, 13:16].

ECV_No Hct_ correlated equally well with clinical markers of disease severity (LV mass index, LVEF, stroke volume index, left atrial area index and NT-pro-BNP) as ECV_Hct_ and partition coefficient, and better than post-contrast T1_myocardium_ (Table 1).

**Table 1:** Correlations between ECV with/without hematocrit, partition coefficient, post contrast T1 myocardium and clinical parameters.

	ECV with Hct	ECV no Hct	Partition Coefficient	Post contrast T1 myocardium
Indexed LV mass	0.48*	0.48*	0.48*	-0.33*

Indexed LA area	0.33*	0.34*	0.33*	-0.30*

LVEF, %	-0.53*	-0.55*	-0.54*	0.34*

Indexed Stroke Volume	-0.46*	-0.47*	-0.48*	0.48*

NT-pro-BNP	0.51*	0.52*	0.48*	-0.34*

*p<0.01

## Conclusions

Native T1_blood_ correlates well with the laboratory-measured values of hematocrit. Our data demonstrates that straight-forward derivation of hematocrit from T1_blood_ can be used as an immediate measure of ECV that may pave its application for nearly instantaneous clinical diagnosis. It remains to be confirmed if the high correlation of ECV_No Hct_ with the conventional calculations may cause blood sampling to become an obsolete complication in clinical practice.

## Funding

TAT and MF are supported by doctoral research fellowships by the National Institute of Health Research (NIHR) and British Heart Foundation, respectively. SKP and MDR are supported by the NIHR Oxford Biomedical Research Centre based at the Oxford University Hospitals Trust at the University of Oxford.

